# Interior and Exterior Decoration of Transition Metal Oxide Through Cu^0^/Cu^+^ Co-Doping Strategy for High-Performance Supercapacitor

**DOI:** 10.1007/s40820-021-00590-x

**Published:** 2021-01-25

**Authors:** Weifeng Liu, Zhi Zhang, Yanan Zhang, Yifan Zheng, Nishuang Liu, Jun Su, Yihua Gao

**Affiliations:** 1 Center for Nanoscale Characterization and Devices (CNCD), School of Physics and Wuhan National Laboratory for Optoelectronics (WNLO), Huazhong University of Science and Technology (HUST), Luoyu Road 1037, Wuhan, 430074 People’s Republic of China; 2 College of Materials Science and Engineering, Guangxi Key Laboratory of Optical and Electronic Materials and Devices, Guilin University of Technology, Guilin, 541004 People’s Republic of China

**Keywords:** Cu^0^/Cu^+^ co-doping, Heterostructure, Transition metal oxide, Supercapacitor

## Abstract

Although CoO is a promising electrode material for supercapacitors due to its high theoretical capacitance, the practical applications still suffering from inferior electrochemical activity owing to its low electrical conductivity, poor structural stability and inefficient nanostructure. Herein, we report a novel Cu^0^/Cu^+^ co-doped CoO composite with adjustable metallic Cu^0^ and ion Cu^+^ via a facile strategy. Through interior (Cu^+^) and exterior (Cu^0^) decoration of CoO, the electrochemical performance of CoO electrode has been significantly improved due to both the beneficial flower-like nanostructure and the synergetic effect of Cu^0^/Cu^+^ co-doping, which results in a significantly enhanced specific capacitance (695 F g^−1^ at 1 A g^−1^) and high cyclic stability (93.4% retention over 10,000 cycles) than pristine CoO. Furthermore, this co-doping strategy is also applicable to other transition metal oxide (NiO) with enhanced electrochemical performance. In addition, an asymmetric hybrid supercapacitor was assembled using the Cu^0^/Cu^+^ co-doped CoO electrode and active carbon, which delivers a remarkable maximal energy density (35 Wh kg^−1^), exceptional power density (16 kW kg^−1^) and ultralong cycle life (91.5% retention over 10,000 cycles). Theoretical calculations further verify that the co-doping of Cu^0^/Cu^+^ can tune the electronic structure of CoO and improve the conductivity and electron transport. This study demonstrates a facile and favorable strategy to enhance the electrochemical performance of transition metal oxide electrode materials.

**Supplementary information:**

The online version of this article (10.1007/s40820-021-00590-x) contains supplementary material, which is available to authorized users.

## Highlights

- A facile strategy is proposed to construct novel CoO and NiO electrode material with interior (Cu^+^) and exterior (metallic Cu^0^) decoration for supercapacitor.
- The co-doped CoO electrode presents four times higher specific capacitance and more cycle stability than that of pristine CoO.
- The conductivity and electron transport rate of CoO with Cu^0^/Cu^+^ co-doping have been effectively improved and confirmed by detailed theoretical calculations.

**Supplementary information:**

The online version of this article (10.1007/s40820-021-00590-x) contains supplementary material, which is available to authorized users.

## Graphical abstract

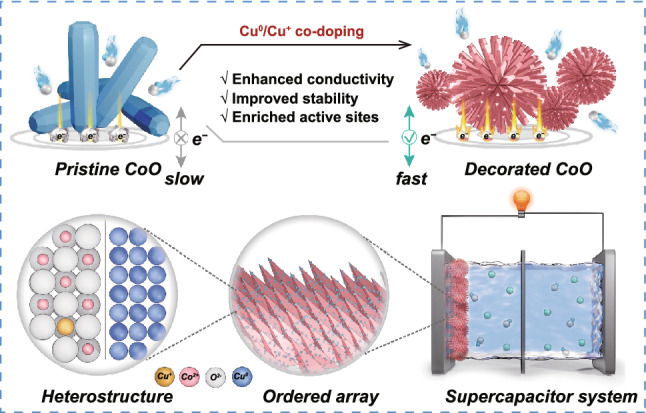

## Introduction

The surging demand for energy storage and conversion has boosted tremendous researches on high-performance electrode materials and devices [1–5]. Supercapacitors (SCs), owing to the merit of fast charge/discharge rate, ultralong life span and high power output, have aroused enormous attentions [6–9]. Carbon materials, conducting polymers and transition metal oxides (TMOs) are the most widely used electrode materials in SCs [10–12]. Particularly, TMOs, such as CoO [13, 14], Co_3_O_4_ [15], NiO [16] and Fe_3_O_4_ [17], deliver much higher specific capacitance than that of carbon materials and conductive polymers benefiting from the multiple reversible faradaic redox reactions.

Among the various TMOs, CoO has attracted more attentions due to high theoretical capacitance than other TMOs (Table S1). However, the stacked structure, poor electronic conductivity and obvious volume change limit its application in SCs. It only shows a low specific capacitance of 100–400 F g^−1^ in practical, which is far below the theoretical value of 4292 F g^−1^ [18, 19]. In order to address these disadvantages, some strategies have been employed, such as coating with carbon materials or conducting polymers to enhance the conductivity and accommodate the large volume change during charge/discharge process [20–22]. However, the synthesis of coating layer is usually complicated [23] and the protection layer often ruptures under drastic volume change [24]. In addition, the intrinsic weak electrical conductivity of TMOs has not been changed.

In this situation, doping of metal or metal cations has been investigated recently and was demonstrated as an effective and simple method to improve the intrinsic electrical conductivity, cycling stability and electrochemical capacitance of TMOs [25–29]. Mai et al. reported that Cu^2+^-doped cobalt–copper carbonate hydroxide could reduce the work function and facilitate charge transfer kinetics [30]. Song et al. reported that doping of metallic Co in CoO can form heterostructures with CoO and significantly increase the electrical conductivity [31]. However, to the best of our knowledge, the co-doping of metal and metal cation in TMOs to exert the synergistic effect of both dopants and to further improve the electrochemical performance of TMOs has not been reported so for. Specifically, Cu, with the second highest electrical conductivity among various metals, is a remarkable candidate for doping [32].

In this study, Cu^0^/Cu^+^ co-doped CoO flower-like nanostructure was synthesized by a facile method and was proved to be a desirable electrode material for SCs. Due to the synergetic effect of Cu^0^/Cu^+^ dopants to effectively adjust the micromorphology and electronic structure of CoO, the specific capacitance and cycling stability have been significantly enhanced. Specifically, the Cu^0^/Cu^+^ co-doped CoO electrode delivers a specific capacitance of 695 F g^−1^ with current density of 1 A g^−1^ and excellent capacitance retention of 93.4% after 10,000 cycles, which are much superior than that of pristine CoO (183.6 F g^−1^ at 1 A g^−1^ and 68.2% after 10,000 cycles). The corresponding asymmetric supercapacitor delivers a high energy density of 35 Wh kg^−1^ at a power density of 800 W kg^−1^. Furthermore, our approach is easily scalable to other TMOs (such as NiO) with much improved electrochemical property. Detailed theoretical calculations indicate that the improved electrochemical performance can be attributed to the Cu^0^/Cu^+^ co-doped CoO with improved intrinsic conductivity and fast charge transfer.

## Experimental Section

### Preparation of Cu^0^/Cu^+^ Co-Doped CoO Nanoflowers

All chemical reagents used are of analytical grade and without any further purification. The Cu^0^/Cu^+^ co-doped CoO is prepared through a typical hydrothermal process. 2 mmol Co(NO_3_)_2_·6H_2_O, 0.2 mmol Cu(NO_3_)_2_·3H_2_O, 6 mmol NH_4_F and 10 mmol urea were added into 70 mL deionized water and stirred for 30 min at room temperature. After that, the prepared solution was sealed into a 100 mL Teflon-liner stainless steel autoclave and placed into an oven at 120 °C for 8 h. After cooling down to room temperature naturally, the precursor was collected with centrifugation, washed with deionized water and alcohol to remove the impurities. Finally, the precursor was transformed into Cu^0^/Cu^+^ co-doped CoO through calcination at 450 °C for 2 h with a heating rate of 2 °C min^−1^ under N_2_ flow. To investigate the effect of metallic Cu (Cu^0^) and Cu^+^-doped concentration on the electrochemical performance of CoO electrode material, different amounts of Cu(NO_3_)_2_·3H_2_O (*M* = 0, 0.1, 0.2, 0.4, 1.0 mmol) and different annealing temperatures (*T* = 350, 450, 600 °C) were systematically studied, abbreviated as CCC-M-T (for example CCC-0.2-450). For comparison, Cu_2_O at the annealing temperatures of 350 °C was prepared by the same procedure without adding of Co(NO_3_)_2_·6H_2_O.

### Preparation of Cu^0^/Cu^+^ Co-Doped NiO Nanosheets

The composite was synthesized via the same method described above, except substituting Co(NO_3_)_2_·6H_2_O with Ni(NO_3_)_2_·6H_2_O. The obtained samples are denoted as CCN-0-450 (pristine NiO), CCN-0.2-400 (Cu^+^-doped NiO) and CCN-0.2-450 (Cu^0^/Cu^+^ co-doped NiO).

### Materials Characterizations

The morphology, crystal structure and chemical composition of samples were investigated using scanning electron microscope (SEM, FEI Nova NanoSEM 450), transmission electron microscopy (TEM, FEI Titan G^2^ 60–300), X-ray diffraction (XRD, Rigaku X-ray diffractometer with Cu Kα radiation) and X-ray photoelectron spectroscopy (XPS, AXIS-ULTRA DLD-600 W). The decomposition process was measured by thermogravimetric analysis (TGA, PerkinElmer Instruments, Pyrisl TGA) under N_2_ flow with the heating rate of 5 °C min^−1^ from 25 to 700 °C. The pore size distribution and specific surface area were tested by N_2_ physisorption at 77 K using the Brunauer–Emmett–Teller (BET, Micomeritics Instrument Corporation, ASAP 2460) method.

### Electrochemical Measurements

The electrochemical testing was performed in a 3 M KOH electrolyte by an electrochemical workstation (Chen Hua CHI660E) at ambient temperature. The cyclic voltammetry (CV) and galvanostatic charge/discharge (GCD) measurements as well as electrochemical impedance spectroscopy (EIS) testing were carried out with a typical three-electrode mode. The as-prepared electrode was used as the working electrode, platinum foil as the counter electrode and Hg/HgO electrode as the reference electrode. EIS measurements were performed in the frequency range from 0.01 Hz to 100 kHz at open circuit potential with 5 mV amplitude. The specific capacitance, energy density and power density are determined by the equations described in the Supporting Information.

### Fabrication of Asymmetric Hybrid Supercapacitor

The quasi-solid asymmetric hybrid supercapacitor (AHSC) was assembled using CCC-0.2-450 electrode as positive electrode and active carbon (AC) as negative electrode, with PVA-KOH gel as electrolyte and cellulose paper as membrane. The electrode and PVA-KOH gel electrolyte were prepared in a typical way [33]. The mass loading for active materials on conductive substrate is in the range of 0.9–1.1 mg cm^−2^ (for example, CCC-0.2-450: 1.0 mg cm^−2^). In order to keep the positive/negative electrode charge balance, the m_+_ (CCC-0.2-450)/m_−_ (AC) should be determined by the following equation:${\text{ C}}_{{{\text{s}} + }} \times m_{ + } \times \, \Delta V_{ + } = {\text{C}}_{{\text{s - }}} \, \times m_{ - } \, \times \, \Delta V_{ - } \,$, where $C_{{\text{s}}}$, $m$, $\Delta V$ are mentioned above.

### Computational Method

The first principles were employed to perform all spin-polarization density functional theory (DFT) calculations within the generalized gradient approximation (GGA) using the Perdew–Burke–Ernzerhof (PBE) formulation [34]. The projected augmented wave (PAW) potentials [35] were chosen to describe the ionic cores and take valence electrons into account using a plane wave basis set with a kinetic energy cutoff of 450 eV. Partial occupancies of the Kohn–Sham orbitals were allowed using the Gaussian smearing method and a width of 0.05 eV. The electronic energy was considered self-consistent when the energy change was smaller than 10^−6^ eV. A geometry optimization was considered convergent when the energy change was smaller than 0.03 eV Å^−1^. In addition, for the Co atoms, the U schemes need to be applied, and the U has been set as 2.7 eV. The formation energies were calculated with the equations described in the Supporting Information.

## Results and Discussion

### Preparation and Characterization of Electrodes

**Scheme 1:**
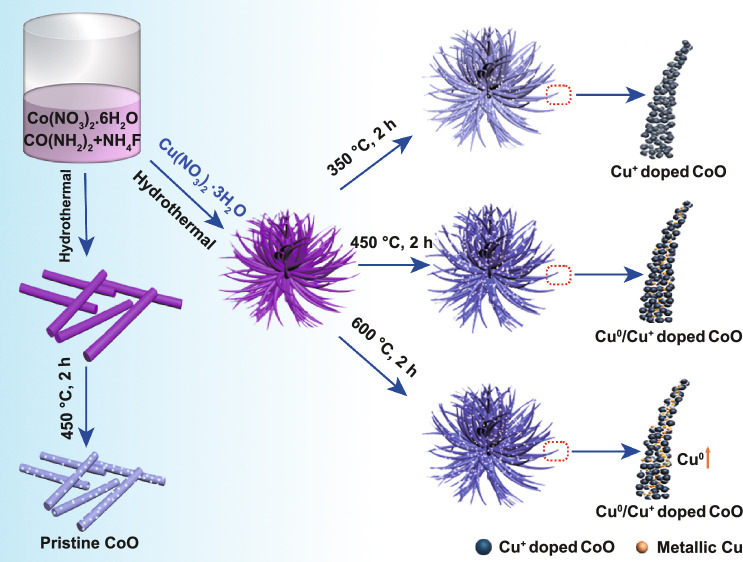
Schematic diagram illustrating the growth process and architecture of CoO without doping, with Cu^+^ doping and with Cu^0^/Cu^+^ co-doping

The schematic illustration for the synthesis of pristine CoO, Cu^+^-doped CoO and Cu^0^/Cu^+^ co-doped CoO is displayed in Scheme 1. Firstly, the copper/cobalt–carbonate–hydroxide precursor was prepared by the co-precipitation reactions between Co(NO_3_)_2_, Cu(NO_3_)_2_, urea and NH_4_F. Then, the precursor was annealed in N_2_ atmosphere to generate 3D flower-like structure with metallic Cu and Cu^+^-doped CoO. However, without the addition of Cu(NO_3_)_2_·3H_2_O, rod-like CoO was obtained. The doping content of Cu^0^ and Cu^+^ can be easily controlled by adjusting the annealing temperature as confirmed by the XRD and TGA analyses shown in Figs. S1 and S2. Specifically, when the annealing temperature is about 350 °C, only Cu^+^-doped CoO nanostructure was obtained. As the temperature increases above 450 °C, the diffraction peaks located at 43.3° and 50.4° corresponding to metallic Cu (PDF No. 04-0836) gradually become stronger as demonstrated by the XRD patterns of CCC-1-450 and CCC-1-600 (The name of different samples is abbreviated as CCC-M-T, in which CCC denotes Cu^0^ or/and Cu^+^-doped CoO, M denotes different additive amount of Cu(NO_3_)_2_·3H_2_O and T denotes different annealing temperatures).

**Fig. 1:**
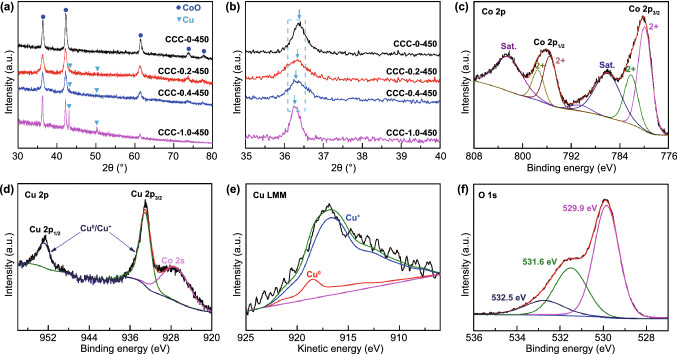
**a**, **b** XRD patterns of the as-prepared CCC-M-450 (*M* = 0, 0.2, 0.4, 1.0) samples. XPS spectra of CCC-0.2-450: **c** Co 2*p*, **d** Cu 2*p*, **e** Cu LMM, **f** O 1*s*

To understand the effect of copper content (0, 0.2, 0.4, 1.0 mmol) on the crystal structure of CoO, the obtained precursors were calcined at the same temperature of 450 °C, and the crystal structures of obtained samples were analyzed by XRD as shown in Fig. 1a. All the samples show similar XRD patterns and the main diffraction peaks are indexed to face-centered cubic CoO (PDF No. 65-2902). In addition, two other diffraction peaks located at 43.3° and 50.4° appear in the sample of CCC-0.2-450, CCC-0.4-450 and CCC-1.0-450 (local magnification shown in Fig. S3), suggesting the existence of cubic metallic Cu. Furthermore, compared with pure CoO (CCC-0-450), the diffraction peaks of (111) plane of CCC-0.2-450, CCC-0.4-450 and CCC-1.0-450 with different Cu doping concentrations are slightly shifted toward lower 2*θ* value (shown in Fig. 1b), which can be attributed to the replacement of Co^2+^ by Cu^+^, leading to the increase in lattice spacing (the ionic radius of Cu^+^ and Co^2+^ is 77 and 74 Å, respectively) [36, 37]. These results indicate that Cu^+^ has been successfully doped in the CoO without changing the crystal structure of CoO.

To further confirm the existence of Cu^0^ and Cu^+^ in the sample, detailed XPS analyses were performed on the CCC-0.2-450. Figure S4 shows the XPS wide-scan survey spectrum, which indicates the existence of Co, Cu and O elements. To understand the detailed bonding information of each element, high-resolution XPS spectra were collected. Figure 1c shows the Co 2*p* spectrum, the binding energy at 779.90/782.15 eV in Co 2*p*_3/2_, and 795.50/797.45 eV in Co 2*p*_1/2_ along with two obviously shakeup satellites (labeled as “Sat.”) can be attributed to Co^2+^, while no peak of Co^3+^ can be observed [30, 38–40]. These results suggest that only CoO exists in the sample. The high-resolution Cu 2*p* spectrum is displayed in Fig. 1d, in which two strong peaks centered at binding energies of 932.91 and 952.71 eV are typically ascribed to Cu^0^/Cu^+^. It should be noted that the peak located at about 926.80 eV corresponds to the Co 2*s*, which can be seen in the wide XPS spectrum shown in Fig. S4. Moreover, no satellite peak of Cu^2+^ (centered at 942.4 eV) is observed in the spectrum, manifesting the only existence of Cu^0^ and Cu^+^ [41, 42]. In addition, the Cu LMM Auger spectrum is further used to distinguish the metallic Cu and Cu^+^ ions (Fig. 1e). Specifically, the peak located at 918.52 eV indicates the existence of Cu^0^, while a broad kinetic energy at 916.18 eV indicates the existence of Cu^+^ [43, 44]. The O 1*s* spectrum (Fig. 1f) shows three characteristic peaks located at 529.9 eV, 531.6 eV and 532.5 eV, which are assigned to M–O–M, M–O–H (oxygen defect sites) and H–O–H bonds [45], respectively. The existence of oxygen defect can be ascribed to the Cu^+^ doping which is beneficial for the electric conductivity [46–48]. These detailed XPS analyses are in good agreement with XRD results and further prove the co-doping of Cu^0^/Cu^+^ in CoO.

**Fig. 2:**
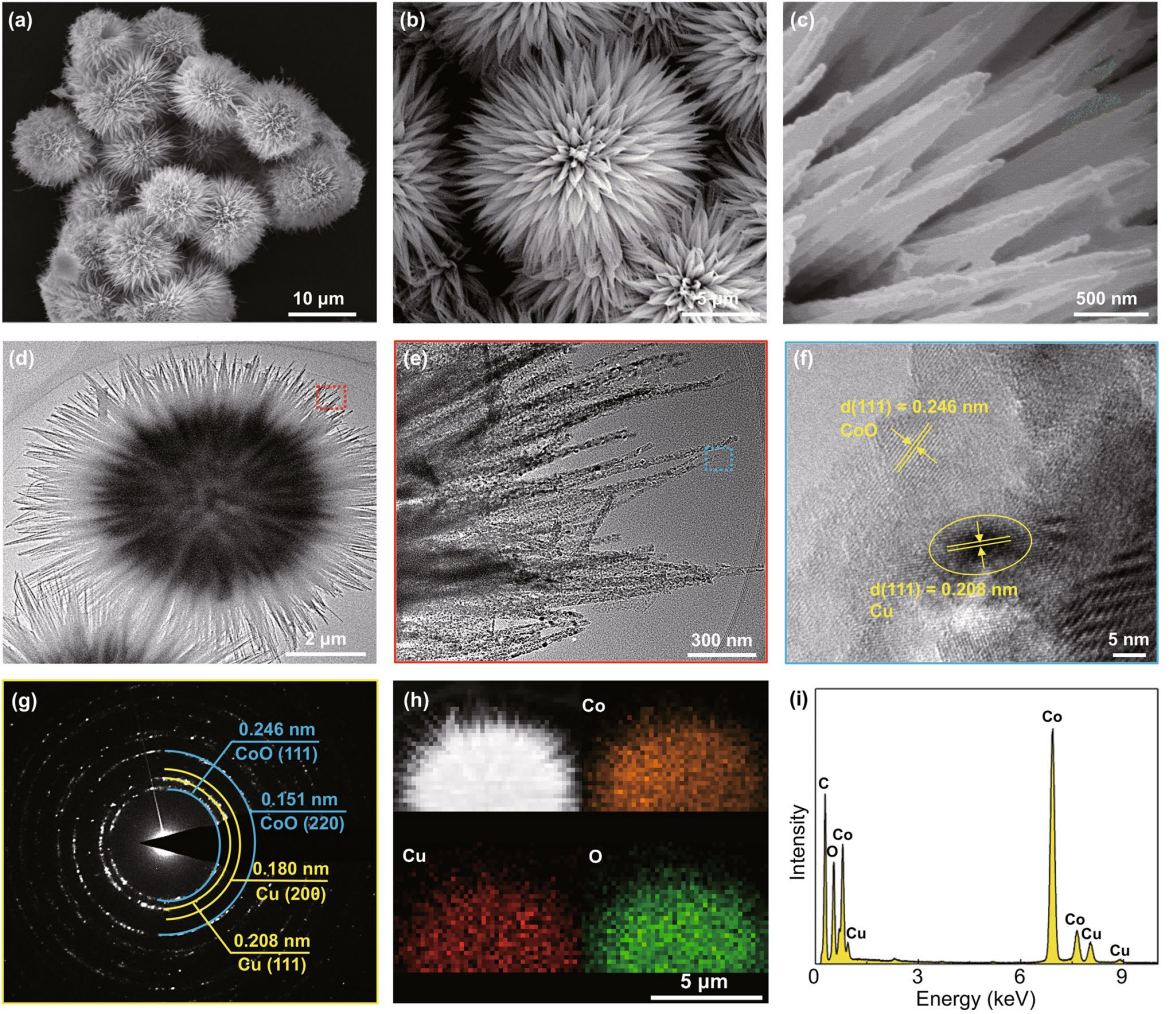
CCC-0.2-450 sample: **a**–**c** Low- and high-magnification SEM images. **d**,** e** bright-field TEM images. **f** High-resolution TEM image. **g** SAED pattern. **h** The corresponding elemental distributions. **i** EDX data of the composite

To understand the morphology of the obtained samples, SEM investigations were performed. Figure 2a–c shows typical SEM images of CCC-0.2-450 taken at different magnifications. It can be observed that the sample has the flower-like morphology, and each flower consists of numerous nanowires radiating out from the center. The effect of Cu doping content on the morphology of CoO has been systematically investigated, and detailed SEM results are shown in Fig. S5. It is found that the Cu doping content has great influence on the morphology of CoO. Specifically, with the increase in copper source, the morphology of CoO evolves from random nanorod to flower-like morphology and finally to nanosheet. The morphology change of CoO can be ascribed to the introduction of moderate copper, offering a trend of one-dimensional growth. However, excessive copper may induce growth to be rather rapidly and excessively, resulting in the nanosheet structure [49]. In addition, the effect of annealing temperature on the morphology of final product was also investigated, and Fig. S6 shows the SEM images of CCC-0.2-350, CCC-0.2-450 and CCC-0.2-600. These detailed SEM results indicate that the annealing temperature has little influence on the morphology of CoO.

Then, detailed microstructure and composition of CCC-0.2-450 were further examined by TEM. Figure 2d shows a typical bright-field TEM image of the sample. It can be noted that each nanoflower consists of numerous one-dimensional nanowires with average length and diameter of 10 and 50 nm, respectively. Figure 2e shows that each nanowire contains numerous interconnected nanoparticles (average size of 25–35 nm) with disordered mesopores, which is beneficial for the penetration of electrolyte ions. High-resolution TEM investigations (Fig. 2f) indicate the formation of mesoporous Cu/CoO heterostructure, in which the lattice fringes of (111) planes of metallic Cu and (111) planes of CoO can be clearly resolved. Selected area electron diffraction (SAED) was used to determine the crystal structure of nanowire as shown in Fig. 2g. Detailed analyses on SAED pattern indicate that each nanowire exhibits polycrystalline nature and can be indexed as metallic Cu and CoO. Energy-dispersive X-ray spectroscopy (EDS) mapping (Fig. 2h) was performed to understand the elemental distributions throughout the whole nanostructure, and the results suggest uniform distribution of Co, Cu and O elements (Fig. 2i). The quantitative analyses on the EDS spectrum suggest the atomic ratio of Co, Cu and O elements to be 45.25:4.36:50.38 (~ 2:0.2:2.2), which is very close to the ratio of reactants. It should be noted that Cu^0^/Cu^+^ co-doped CoO sample was dispersed on carbon-coated Mo TEM grid rather than normal Cu TEM grid for the structure and composition characterizations.

### Electrochemical Performance and Kinetics Analysis

**Fig. 3:**
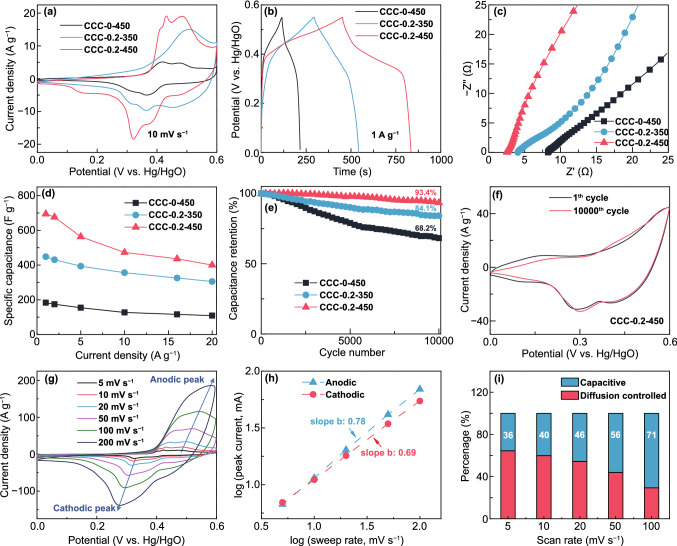
Comparison of electrochemical performances of pristine CoO (CCC-0-450), Cu^+^-doped CoO (CCC-0.2-350), Cu^0^/Cu^+^ co-doped CoO (CCC-0.2-450) in a three-electrode configuration: **a** CV curves, **b** GCD curves, **c** EIS plots, **d** specific capacitance, **e** cycling performance at 50 mV s^−1^, **f** the 1st and the 10000th cycle CV plot of CCC-0.2-450 electrode. **g**–**i** CV curves, linear relation between log (*v*) and log (*i*), and the contribution ratio of capacitive and diffusion-controlled process in CCC-0.2-450

The electrochemical performance of the co-doped samples and reference samples was first evaluated in a three-electrode cell with the working potential window from 0 to 0.6 V (vs. Hg/HgO) at room temperature. Figure 3a shows the CV curves of pristine CoO (CCC-0-450), Cu^+^-doped CoO (CCC-0.2-350) and Cu^0^/Cu^+^ co-doped CoO (CCC-0.2-450) electrodes at the scan rate of 10 mV s^−1^. All the samples show a distinct redox peak, indicating the capacitance is mainly based on the redox mechanism. It can be observed that the Cu^0^/Cu^+^ co-doped CoO exhibits a larger integral area in the CV curve than that of other samples, indicating a much superior capacitance. In addition, three pairs of redox peaks appear in CCC-0.2-450 and CCC-0.2-350, different from only two pairs of redox peaks in pristine CoO. This newly emerged redox peaks further confirm that copper ion has been successfully doped in CoO and participated in redox reactions. The slight shift of redox peaks is suggested to be related to different annealing temperatures [50, 51]. The GCD curve shown in Fig. 3b indicates that the Cu^0^/Cu^+^ co-doped CoO electrode shows much longer discharging time than the other two samples at the same current density of 1 A g^−1^, suggesting the co-doping of metallic Cu and Cu^+^ can significantly enhance the electrochemical capacitance.

In order to elucidate the impedance and charge transfer kinetics influenced by the synergistic effect of Cu^0^ and/or Cu^+^ doping, the Nyquist plots of EIS measurements were investigated as shown in Figs. 3c and S7 (the magnified plot and the equivalent circuit). Obviously, the CCC-0.2-450 electrode exhibits the lowest internal resistance (*R*_s_: 2.53 Ω) and the lowest interfacial charge transfer resistance (*R*_ct_: 2.66 Ω) than the other two electrodes (detailed data shown in Table S2). Meanwhile, the straight line in the low-frequency region shows quasi-vertical feature, demonstrating low Warburg resistance for fast ions diffusion [52]. The reduced resistance can be attributed to the fact that the doping of Cu^+^ ions is beneficial for improving the intrinsic electric conductivity of CoO while the conductive metallic Cu provides an “expressway” for electron transport.

Based on the GCD curves (shown in Fig. S8), the specific capacitance of the three electrodes was calculated and compared in Fig. 3d. The CCC-0-450, CCC-0.2-350 and CCC-0.2-450 electrodes exhibit maximum specific capacitance of 184, 448 and 695 F g^−1^ at a current density of 1 A g^−1^, respectively. In addition, 58% of the capacitance is retained when the current density increases to 20 times (20 A g^−1^). Moreover, different annealing temperatures (CCC-0.2-350, CCC-0.2-450, CCC-0.2-600) and the optimal Cu doping content (CCC-0.1-450, CCC-0.2-450, CCC-0.4-450 and CCC-1.0-450) were investigated, and the results are shown in Figs. S8–S10. Both the CV and GCD curves indicate that CCC-0.2-450 sample possesses the optimal electrochemical performance. The decrease in capacitance can be ascribed to the impeded redox reactions between CoO and electrolyte [23] and the little capacitance contribution of Cu due to the excessive Cu doping. (The electrochemical performance of Cu_2_O is shown in Fig. S11).

In addition, long-term cycling test was further examined and the results are shown in Fig. 3e. The CCC-0.2-450 (Cu^0^/Cu^+^ co-doped CoO) electrode also exhibits satisfactory cycling performance, and 93.4% of the initial capacitance is maintained after 10,000 cycles, which is higher than the value of 68.2% for pristine CoO. Moreover, it is worth mentioning that the CV shape (Fig. 3f) and morphology (Fig. S12) of the CCC-0.2-450 electrode materials after 10,000 cycles are almost the same with the first cycle, further confirming the highly stable cycling performance. In particular, this co-doped electrode also demonstrates higher performance than many recently reported researches on Co-based oxide (shown in Table S3).

To better understand the energy storage mechanism, the electrochemical reaction kinetics of Cu^0^/Cu^+^ co-doped CoO (CCC-0.2-450) electrode were further studied. Firstly, the diffusion-controlled and the surface capacitance contribution can be identified by analyzing the CV curves (Fig. 3g) [21]. As shown in Fig. 3h, the calculated constant *b* values (details in Supporting Information) of CCC-0.2-450, which are related to different charge storage mechanisms, are 0.78 and 0.69 for the anodic peak around 0.5 V and cathodic peak around 0.3 V, respectively. These results indicate that the charge storage includes both diffusion-controlled process and surface-controlled capacitive process. In addition, the different charge storage contributions can be quantitatively estimated (details in Supporting Information) [53, 54]. As shown in Figs. 3i and S13, when the scan rates increase from 5 to 100 mV s^−1^, the capacitive contribution increases from 36% to 71%, implying the surface capacitive behavior dominates the charge storage mechanism when the scan rate is higher than 50 mV s^−1^.

### Structure and Performance of NiO Used Co-Doped Strategy

**Fig. 4:**
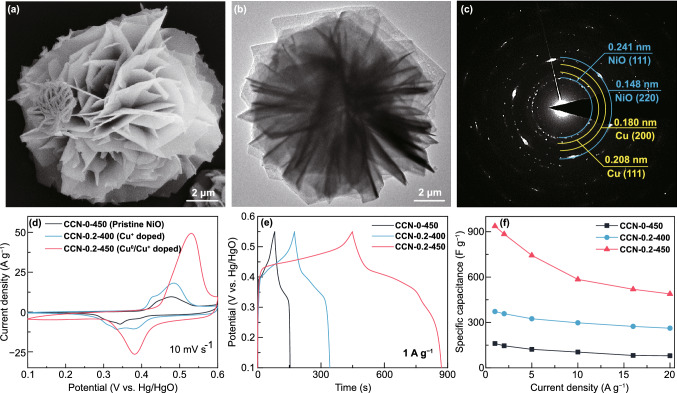
Cu^0^/Cu^+^ co-doped NiO (CCN-0.2-450) sample: **a** SEM image, **b** bright-field TEM image, **c** electron diffraction pattern. Comparison of electrochemical performance of pristine NiO, Cu^+^-doped NiO, and Cu^0^/Cu^+^ co-doped NiO in a three-electrode configuration: **d** CV curves, **e** GCD curves, **f** specific capacitance

Furthermore, to verify the universality of this novel method, the Cu^0^/Cu^+^ co-doping strategy was applied to other TMOs (NiO), and the electrochemical performance was also investigated. Detailed XRD analyses (Fig. S14a) on the prepared samples indicate that both the Cu^+^ doping and Cu^0^/Cu^+^ co-doping can be successfully achieved in NiO. The morphology and microstructure of the Cu^0^/Cu^+^ co-doped NiO (CCN-0.2-450 sample) are exhibited in Fig. 4a–c. The SEM image shows the flower-like structure formed by many nanosheets (Fig. 4a), which is consistent with the TEM observations shown in Fig. 4b. The SAED analysis (Fig. 4c) and the high-resolution TEM image (Fig. S14b) indicate the existence of metallic Cu and NiO. The CV analysis (Fig. 4d) and GCD analysis (Fig. 4e) were carried out to compare the electrochemical performance of the pristine NiO (CCN-0-450), Cu^+^-doped NiO (CCN-0.2-400) and Cu^0^/Cu^+^ co-doped NiO (CCN-0.2-450). The results demonstrate that the Cu^0^/Cu^+^ co-doped NiO presents the highest area under the CV curve and the longest discharging time under the GCD curve, indicating the maximum energy storage capacitance. The specific capacitance of three different electrodes at various current density is compared in Figs. 4f and S15. The Cu^0^/Cu^+^ co-doped NiO shows the highest capacitance of 936 F g^−1^, and 52% of the capacitance was retained as the current density extends to 20 A g^−1^ (CCN-0-450 with 161 F g^−1^ at 1 A g^−1^ and 49% capacitance was retained at 20 A g^−1^). These results further prove the feasibility of this facile co-doping strategy to achieve superior electrochemical performance due to strong synergy effect of Cu^+^ and metallic Cu co-doping in NiO.

### Electrochemical Properties of AHSC Devices

**Fig. 5:**
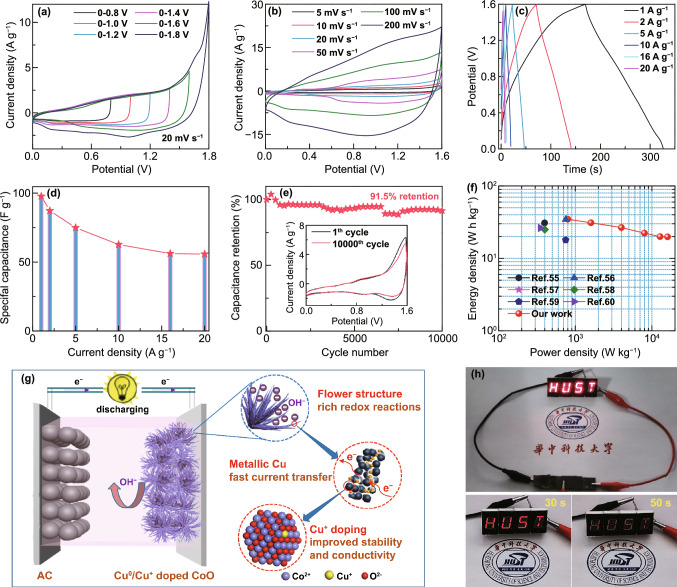
Electrochemical performance of Cu^0^/Cu^+^ co-doped CoO//AC AHSC device: **a** CV curves at different potential windows. **b** CV curves at different scan rates. **c** GCD curves. **d** Specific capacitance. **e** Cycling performance (inset: CV curves of the first and the last cycle). **f** Ragone plot. **g** Schematic of Cu^0^/Cu^+^ co-doped CoO electrode. **h** Symbols of “HUST” lighted by our device

To evaluate the practical application of the fabricated Cu^0^/Cu^+^ co-doped CoO electrode for energy storage, an asymmetric hybrid supercapacitor (AHSC) device was constructed. The CV curves of Cu^0^/Cu^+^ co-doped CoO electrode (0–0.6 V) and AC electrodes (− 1 to 0 V) with the scan rate of 10 mV s^−1^ are shown in Fig. S16. Figure 5a indicates that the maximal operation voltage of the device can extend to 1.6 V without significant polarization effect. The CV curves of the as-prepared device in a potential window of 0 to 1.6 V are shown in Fig. 5b. Benefiting from the combined contribution of faradic reaction electrode (CCC-0.2-450) and capacitive electrode (AC), the CV curves show the characteristics of both pseudocapacitance and EDLC. Furthermore, even when the scan rate increases to 200 mV s^−1^, the curve shows no distortion, which indicates good rate capability and reversibility. Accordingly, the GCD curves (Fig. 5c) with different current densities show high coulombic efficiency, demonstrating good capacitive behavior and electrochemical reversibility. The specific capacitance of the AHSC device was calculated to be 97.7 F g^−1^ at 1 A g^−1^ (Fig. 5d), and 57% of its initial specific capacitance was retained even at 20 A g^−1^ (55.8 F g^−1^), further proving the good rate performance. The cycling performance of the device was tested at a high scan rate of 50 mV s^−1^, exhibiting excellent cycling stability with ~ 91.5% capacitance retention even after 10,000 cycles (Fig. 5e and the inset).

The energy density and power density are essential practical figure-of-merits. Based on the large capacitance and high voltage, the calculated Ragone plots of the device are presented in Fig. 5f. Impressively, our device delivers a maximum energy density of 34.7 Wh kg^−1^ at 800 W kg^−1^ and can still remain 19.8 Wh kg^−1^ at a power density of 16 kW kg^−1^. It is worth noting that the energy density in this work was more superior than other similar reported devices employing Co-based oxides [55–60]. With the working potential of 1.6 V and the merit of high power density in our device, a series of red light-emitting diodes (LEDs) were lighted for 50 s as shown in Fig. 5h.

Based on these detailed results, the working principle and mechanism for the outstanding electrochemical performance of Cu^0^/Cu^+^ co-doped CoO electrode are illustrated in Fig. 5g. (1) First and foremost, the doping of small amount of Cu^+^ ions can improve the inherent electrical conductivity and electrochemical activity of CoO and enhance redox reaction kinetics. Meanwhile, the highly conductive Cu^0^ metal forms heterostructure with CoO, which serves as a “superhighway” for electron transfer to the conductive substrate [61]. (2) Small amount of Cu^+^ ions doping in CoO was suggested to prevent the active materials from losing mechanical integrity [62]. In addition, the formation of alternately linked metallic Cu^0^ and CoO nanocrystals in nanowires can relax the volume change during the repeated charge/discharge processes. Therefore, the synergetic effect of Cu^0^/Cu^+^ co-doping in CoO can improve the cycling life span of CoO electrode. (3) The co-doping of Cu^0^/Cu^+^ leads to the formation of 3D flower-like structure with interconnected mesoporous nanowire arrays in Cu^0^/Cu^+^ co-doped CoO, contributing to the high-specific surface area (83.5 m^2^ g^−1^) and unique mesoporous architecture (results shown in Fig. S17). This specific area is higher than that of pristine CoO with nanorod structure (33.1 m^2^ g^−1^ for CCC-0-450) and is also higher than that of heavily Cu-doped CoO with nanosheet structure (57.2 m^2^ g^−1^ for CCC-1.0-450). In this situation, such structural architecture can facilitate the electrolyte penetration and shorten the ion diffusion path and hence leads to the increased specific capacitance.

### DFT Calculations

**Fig. 6:**
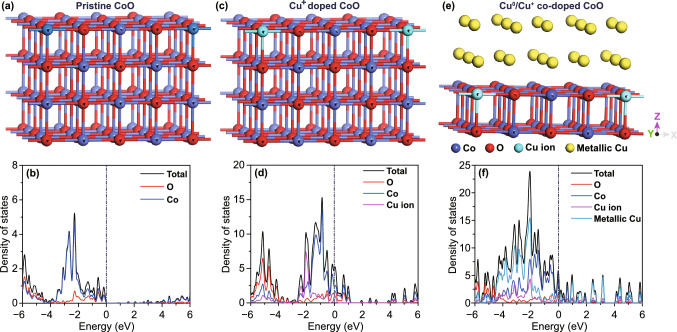
Atomic structure models and DFT calculation of the DOS for **a**, **b** pristine CoO, **c**, **d** Cu^+^-doped CoO and **e**, **f** Cu^0^/Cu^+^ co-doped CoO

To further understand the effect of Cu^+^ doping and Cu^0^/Cu^+^ co-doping on the electronic structure of CoO, and hence on the electrochemical performance of CoO electrode materials, theoretical calculations were performed and the results are shown in Fig. 6. Figure 6a–f displays the atomic structure models of pristine CoO, Cu^+^-doped CoO and Cu^0^/Cu^+^ co-doped CoO, and the corresponding calculated density of states (DOS). It can be noted that through the doping of Cu^+^, the electrons show continuous occupied states at the Fermi level and some impurity level appears in the conduction band, which reduces the energy barrier for the electron transition (Fig. 6b, d) and leads to enhanced electrical conductivity of CoO. In addition, after the formation of heterostructure between metallic Cu and Cu^+^-doped CoO, more impurity level is shown in the conduction band (Fig. 6f), implying further enhanced electrical conductivity and faster electron transport way. Benefiting from the improved electrical conductivity, the electrons generated from the redox reactions can be transferred rapidly to the current collector. The increased electronic mobility leads to enhanced reaction kinetics and improved electrochemical performance. Moreover, the formation energies of Cu^+^-doped CoO (− 2.37 eV) and Cu^0^/Cu^+^ co-doped CoO (− 3.92 eV) were calculated, which implies the as-prepared electrode is more stable after the doping process and the formation of heterostructure. These theoretical calculation results verify that the introduction of appropriate amount of metallic Cu and Cu^+^ ion can effectively enhance the intrinsic electrical conductivity and electron transport, which also agree with the EIS results discussed above.

## Conclusion

In summary, we reported the rational design and fabrication of a Cu^0^/Cu^+^ co-doped CoO electrode with much improved electrochemical performance. Benefiting from the merits of flower-like structure with plentiful active sites, metallic Cu heterostructure with fast electron transfer path and as well as Cu^+^-doped CoO with enhanced inherent electrical conductivity, optimized Cu^0^/Cu^+^ co-doped CoO shows exceptional electrochemical performance, including high specific capacitance, good rate capability and long-term cycling durability. Furthermore, the fabricated asymmetric hybrid supercapacitors also exhibit outstanding energy/power density and excellent electrochemical stability. This co-doping strategy is also applicable to other TMOs, such as Cu^0^/Cu^+^ co-doped NiO with enhanced electrochemical performance. Our facile approach provides a favorable strategy to synthesize high-performance TMOs-based electrode materials for next-generation energy storage devices.

## Supplementary information

Below is the link to the electronic supplementary material.

Supplementary information (PDF 2108 kb)
