# Supplementary material for: Interior and Exterior Decoration of Transition Metal Oxide Through Cu0/Cu+ Co-Doping Strategy for High-Performance Supercapacitor
Source: Nanomicro Lett. 2021 Jan 25;13:61. doi: 10.1007/s40820-021-00590-x (PMC8187495; doi:10.1007/s40820-021-00590-x)
Supplement: Supplementary file 1 — Supplementary information (PDF 2108 kb) [file 40820_2021_590_MOESM1_ESM.pdf]

Supporting Information for

Interior and Exterior Decoration of Transition Metal Oxide through Cu^0/Cu^+ Co-Doping Strategy for High-Performance Supercapacitor

Weifeng Liu[1], Zhi Zhang[1, *], Yanan Zhang[1], Yifan Zheng[1], Nishuang Liu[1], Jun Su[1], Yihua Gao[1, 2, *]

[1]Center for Nanoscale Characterization & Devices (CNCD), School of Physics & Wuhan National Laboratory for Optoelectronics (WNLO), Huazhong University of Science and Technology (HUST), Luoyu Road 1037, Wuhan 430074, P.R. China

[2]College of Materials Science and Engineering, Guangxi Key Laboratory of Optical and Electronic Materials and Devices, Guilin University of Technology, Guilin 541004, China

*Corresponding author E-mail: **zzhang@hust.edu.cn (Z. Zhang); gaoyihua@hust.edu.cn (Y. H. Gao)**

Supplementary Tables and Figures

Calculation of specific capacitance, energy density, and power density

The specific capacitance, energy density, and power density are determined by the following calculation formulas:

$$C_s = \frac{I \times \Delta t}{m \times \Delta V} \tag{1}$$

$$E = \frac{1}{2 \times 3.6} C_s \Delta V^2 \tag{2}$$

$$P = \frac{3600 \times E}{\Delta t} \tag{3}$$

Where C_s ($F\ g^{-1}$) is the specific capacitance, I (A) is the discharge current, Δt (s) is the discharge time, m (g) is the full mass of the active election, ΔV (V) is the operating voltage range during a full discharge, E ($Wh\ kg^{-1}$) and P ($W\ kg^{-1}$) is the energy density and power density, respectively.

Determination of diffusion-controlled and surface capacitance contribution

In theory, diffusion-controlled and surface capacitance contribution can be identified by analyzing the CV curves according to the equation (4) and (5):

$$i = av^b \tag{4}$$

$$\log i = b\log v + \log a \tag{5}$$

where i and v is the current response and scan rate; a and b are the adjustable constants. The b value, estimated from the slope of the linear fit from $\log i$ versus $\log v$, is related to different charge storage mechanism. When b approaches 0.5, the current is controlled by diffusion-controlled behavior; while b is close to 1, the current is dominated by the capacitive-type charge storage behavior.

In addition, the different charge storage contributions can also be quantitatively estimated using the following equation:

$$i(V) = k_1 v + k_2 v^{0.5} \tag{6}$$

$$\frac{i(V)}{v^{0.5}} = k_1 v^{0.5} + k_2 \tag{7}$$

where i is the response current at a specific potential, v is the scan rate. $k_1 v$ and $k_2 v^{0.5}$ are capacitive and diffusion-controlled currents, respectively.

Calculations of formation energy

The formation energies (FEs) were calculated as FEs= Etotal -μCu -ECoO+μCo , where Etotal is the total energies of Cu doping structure, and μCu , μCo is the Cu, Co chemical potential. ECoO is the energy of CoO structure, respectively. For the heterojunction structure, the formation energies can be obtained by: FEs=EA/B-EA-EB, where EA/B is the energy of heterojunction structure, EA is the energy of Cu doping CoO slab and EB is the energy of Cu metal slab.

Table S1 Comparison the theoretical capacity of typical TMOs

Materials	Molecular weight (g mol^{-1})	Chemical reaction equation	Potential window (V)	Theoretical capacity (F g^{-1})
**CoO**	**74.9**	**$CoO + 2OH^- \leftrightarrow CoO_2 + H_2O + 2e^-$**	**0.6**	**4292**
Co_3O_4	240.8	$Co_3O_4 + 4OH^- \leftrightarrow 3CoO_2 + 2H_2O + 4e^-$	0.45	3561
NiO	74.7	$NiO + 3OH^- \leftrightarrow NiOOH + H_2O + e^-$	0.5	2583
MnO_2	86.9	$MnO_2 + Na^+ + e^- \leftrightarrow NaMnO_2$	0.8	1387
Fe_2O_3	159.7	$Fe_2O_3 + 3H_2O + 6e^- \leftrightarrow 2Fe + 6OH^-$	1.0	3625

The theoretical capacity calculated based on the following formula:

$$C_s = \frac{n \times F}{m \times v}$$

Where n is the electron number, F is the Faraday constant (96,485 $C\ mol^{-1}$), m is the molecular weight and v is the redox potential window.

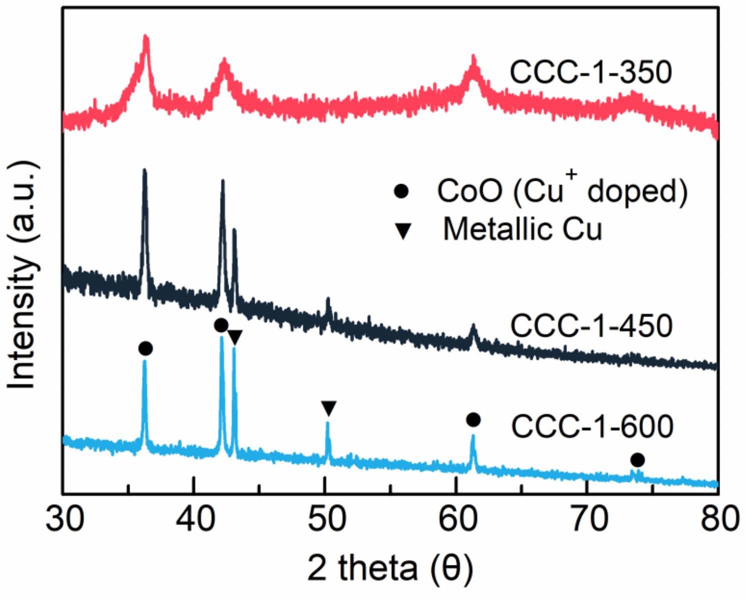

Fig. S1 XRD patterns of the CCC-1-350, CCC-1-450 and CCC-1-600

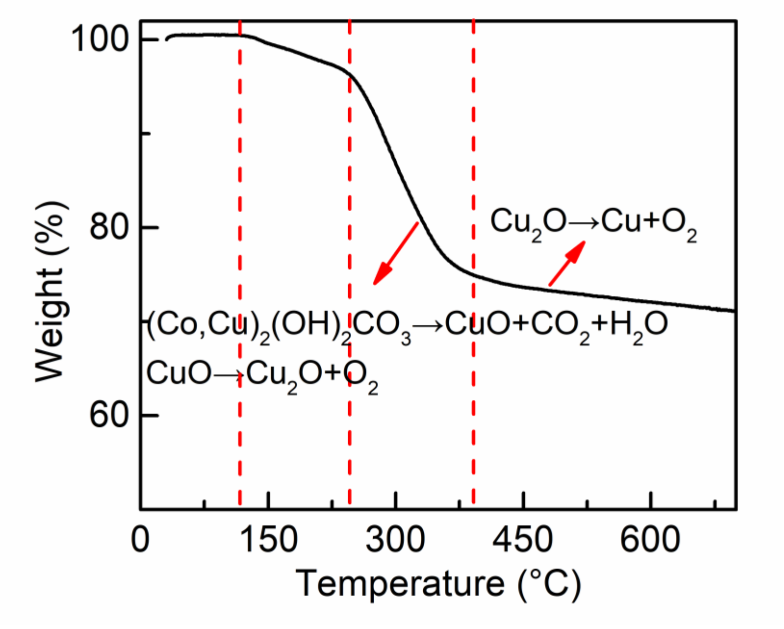

Fig. S2 TG curve of the CCC-0.2-450 under the N_2 atmosphere

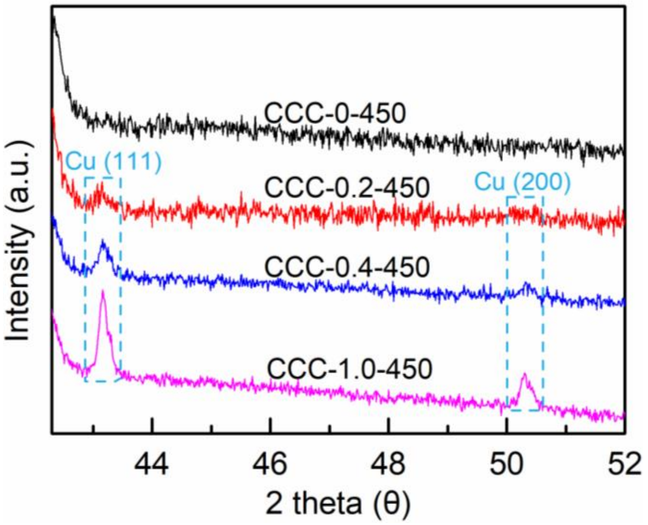

Fig. S3 Local magnification on the XRD patterns of the CCC-0-450, CCC-0.2-450 CCC-0.4-450 and CCC-1.0-450

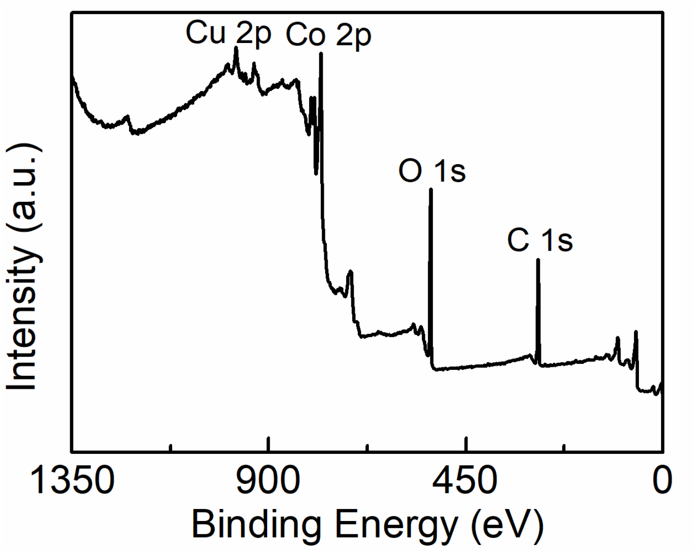

Fig. S4 The wide XPS spectrum of CCC-0.2-450

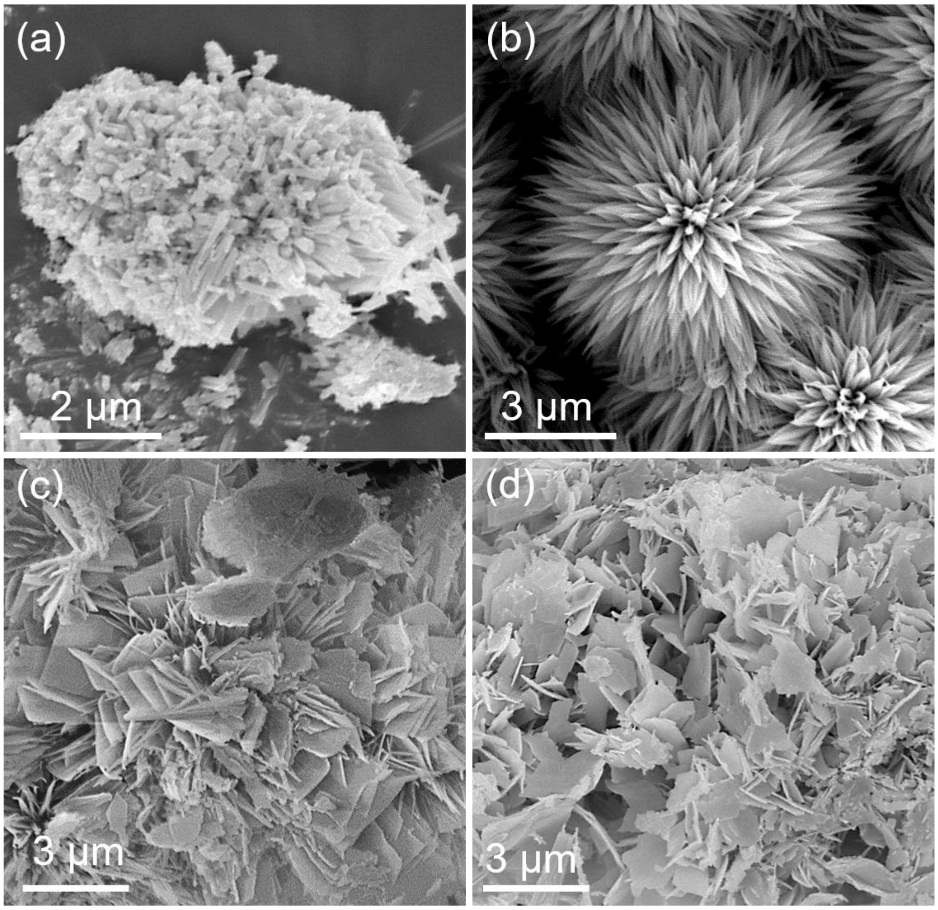

Fig. S5 SEM images of **a** CCC-0-450, **b** CCC-0.2-450, **c** CCC-0.4-450 and **d** CCC-1.0-4

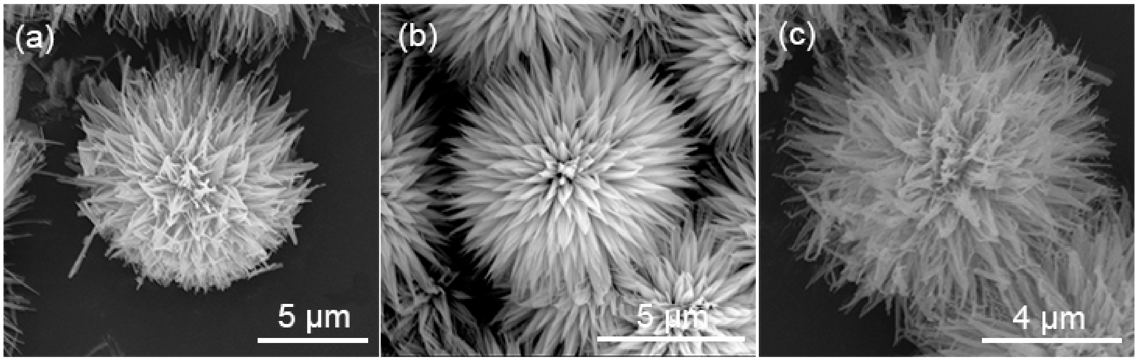

Fig. S6 SEM images of **a** CCC-0.2-350, **b** CCC-0.2-450 and **c** CCC-0.2-600

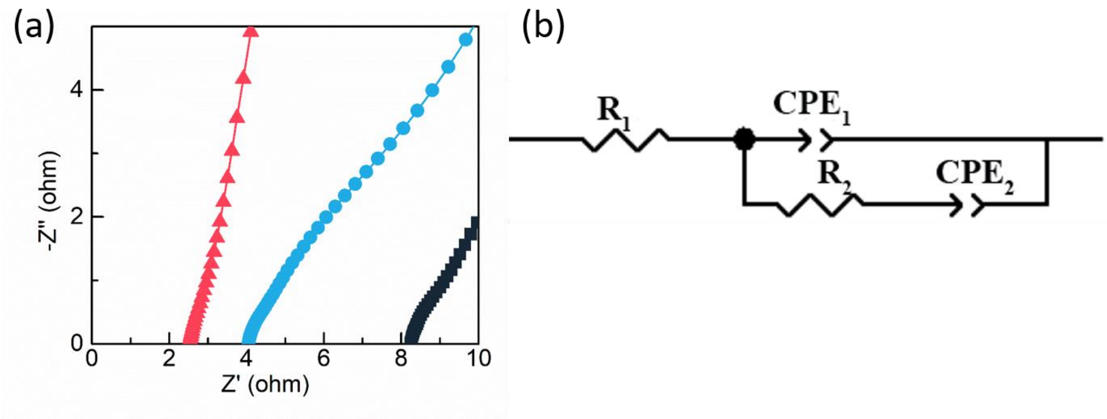

Fig. S7 a The magnified high frequency region of the Nyquist plot, **b** Equivalent circuit for the fitting of CCC-0.2-450 EIS spectra. R_1 represent bulk solution resistance ($R_{s,}$), R_2 represent charge transfer resistance (R_{ct}), CPE_1 and CPE_2 represent pseudocapacitance and constant phase element, respectively

Table S2 Resistance of the samples by the fitting of the EIS spectra

Materials	R_s (Ω)	R_{ct} (Ω)
**CCC-0.2-450**	**2.534**	**2.662**
**CCC-0.2-350**	3.976	3.717
**CCC-0-450**	8.176	6.965

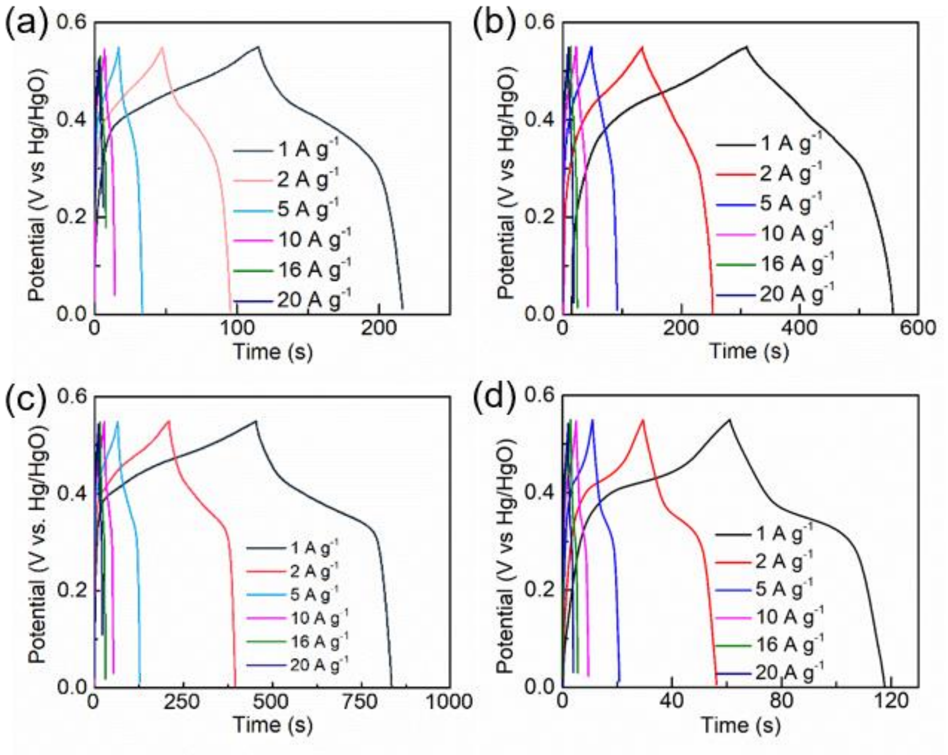

Fig. S8 GCD curves: **a** CCC-0-450 (Pristine CoO), **b** CCC-0.2-350 (Cu^+ doped CoO), **c** CCC-0.2-450 (Cu^0/Cu^+ co-doped CoO), **d** CCC-0.2-600 (Cu^0/Cu^+ co-doped CoO)

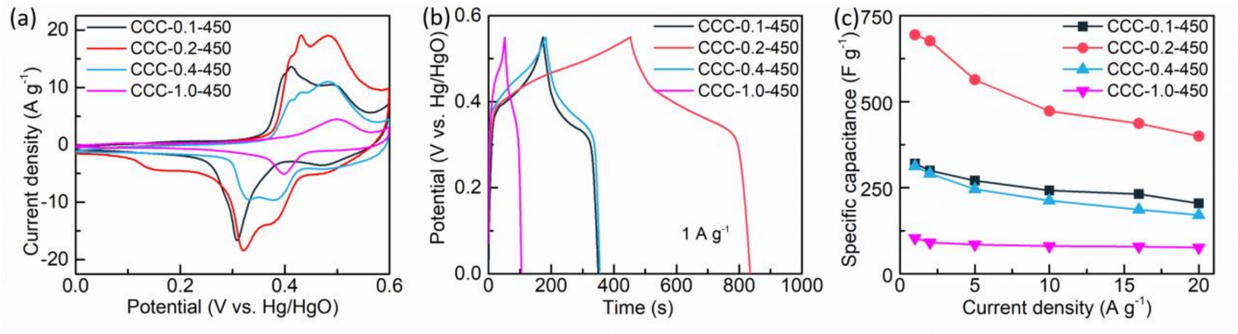

Fig. S9 **a** CV curves at 10 mV s^{-1}, **b** GCD curves at 1 A g^{-1}, and **c** the specific capacitance at different Cu doping content of CCC-0.1-450, CCC-0.2-450, CCC-0.4-450 and CCC-1.0-450

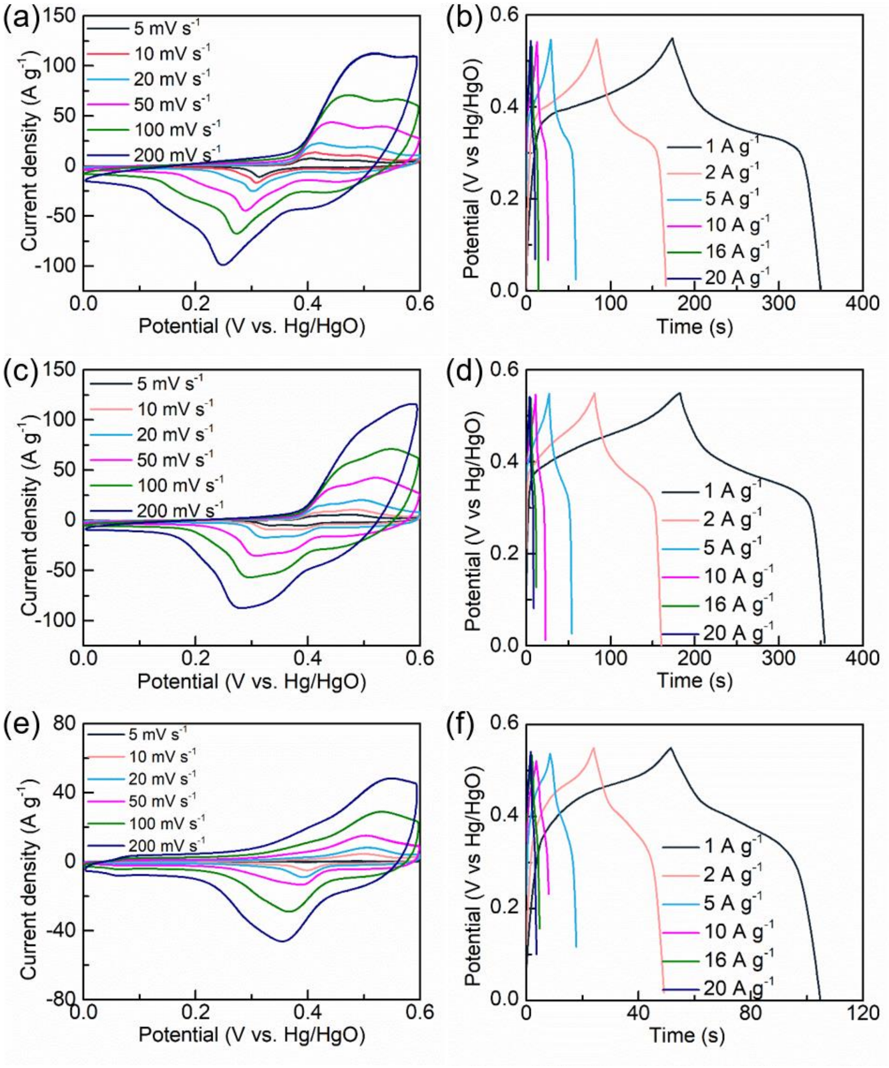

Fig. S10 CV curves **a** and GCD curves **b** of CCC-0.1-450; CV curves **c** and GCD curves **d** of CCC-0.4-450; CV curves **e** and GCD curves **f** of CCC-1.0-450

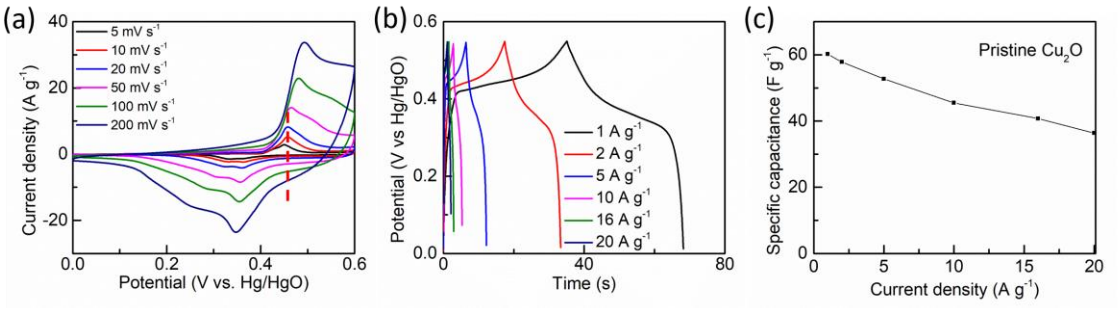

Fig. S11 Cu_2O at the annealing temperature of 350 °C: **a** CV curves, **b** GCD curves and **c** the specific capacitance

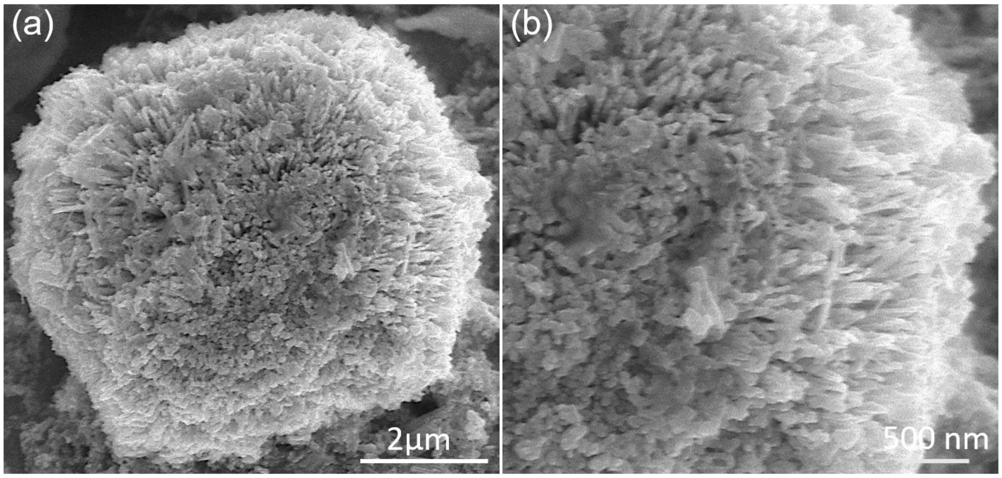

Fig. S12 SEM images of CCC-0.2-450 electrode after 10000 cycles

Table S3 Comparison of electrochemical performance of CCC-0.2-450 electrode with previously reported electrodes

Electrode material	Specific capacity (F g^{-1})	Capacitance maintains	Year	Ref.
**Cu^0/Cu^+ co-doped CoO (CCC-0.2-450)**	**695 (1 A g^{-1})**	**93.4% (10000 cycles)**		**Our work**
porous CoO nanowall	352 (1 A g^{-1})	92.9% (5000 cycles)	2019	[S1]
manganese doped Co_3O_4	668.4 (1 A g^{-1})	104% (10000 cycles)	2019	[S2]
CoO nanocones	319.5 (1 mA cm^{-2})	92.6% (5000 cycles)	2018	[S3]
Co_3O_4 nanosheets@ nitrogen-doped carbon	581 (1 A g^{-1})	95.2% (5000 cycles)	2018	[S4]
CoO/C Composite	648 (0.5 A g^{-1})	100% (8000 cycles)	2017	[S5]
nano-Co_3O_4 decorated with gold	681 (0.5 A g^{-1})	83.1% (13000 cycles)	2017	[S6]
Co_3O_4/NGF composite	451 (1 A g^{-1})	95% (1000 cycles)	2015	[S7]
ball milled CoO	600 (0.5 A g^{-1})	95.3% (2000 cycles)	2014	[S8]
graphene/Co_3O_4 nanocrystals	570 (1 A g^{-1})	83.4% (5000 cycles)	2020	[S9]

Co_3O_4 grow on porous carbons	659.7 (10 mV s^{-1})	87.5% (2000 cycles)	2020	[S10]
hydroxyl-rich Co_3O_4	~502 (1 A g^{-1})	99.7% (5000 cycles)	2020	[S11]
oxygen-vacancy Co_3O_4/Graphene	978.1 (1 A g^{-1})	99.3% (20000 cycles)	2018	[S12]
Co_3O_4@biomass-derived carbon fiber@Co_3O_4	892 (1 A g^{-1})	88% (6000 cycles)	2018	[S13]

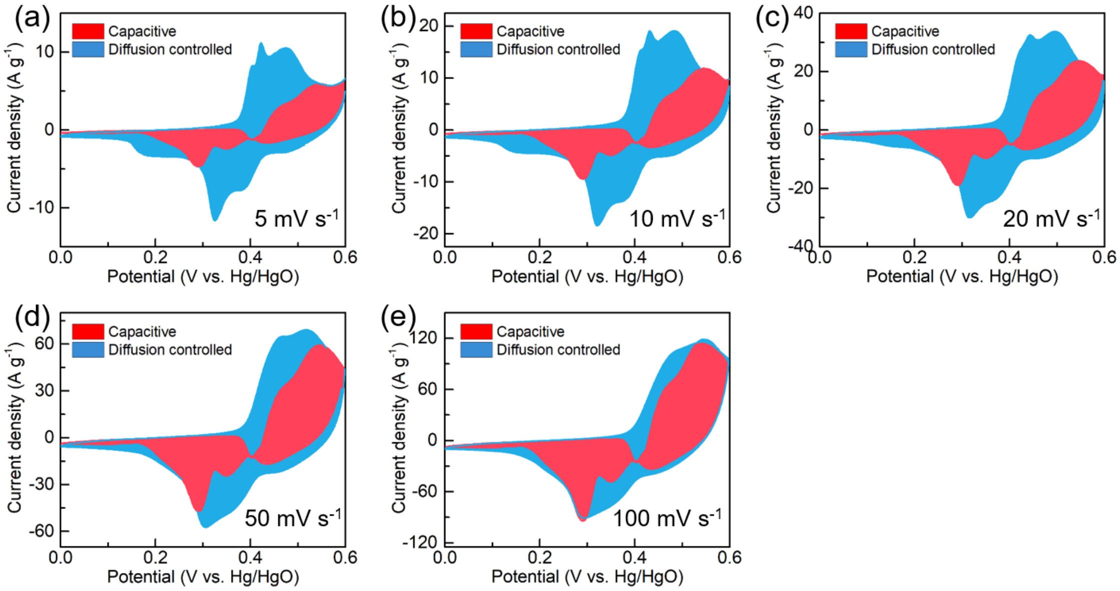

Fig. S13 Contribution ratio of capacitive and diffusion controlled: **a** 5 mV s^{-1}, **b** 10 mV s^{-1}, **c** 20 mV s^{-1}, **d** 50 mV s^{-1}, **e** 100 mV s^{-1}

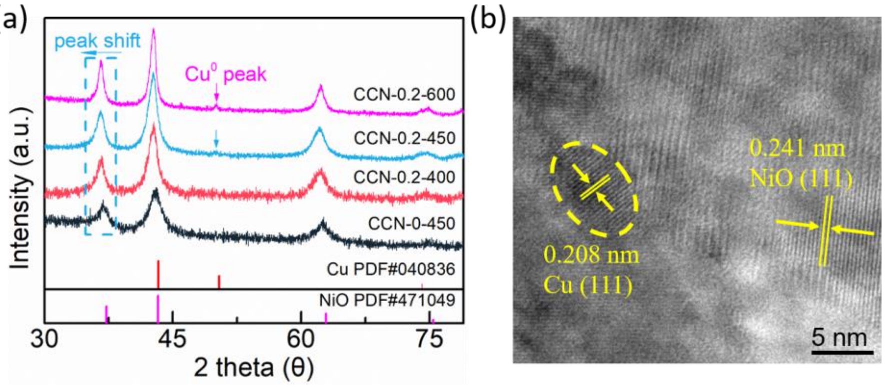

Fig. S14 a XRD patterns of the as-prepared CCN-0-450 (pristine NiO), CCN-0.2-400 (Cu^+ doped NiO), CCN-0.2-450 (Cu^0/Cu^+ co-doped NiO) and CCN-0.2-600 (Cu^0/Cu^+ co-doped NiO); **b** High-resolution TEM image of CCN-0.2-450

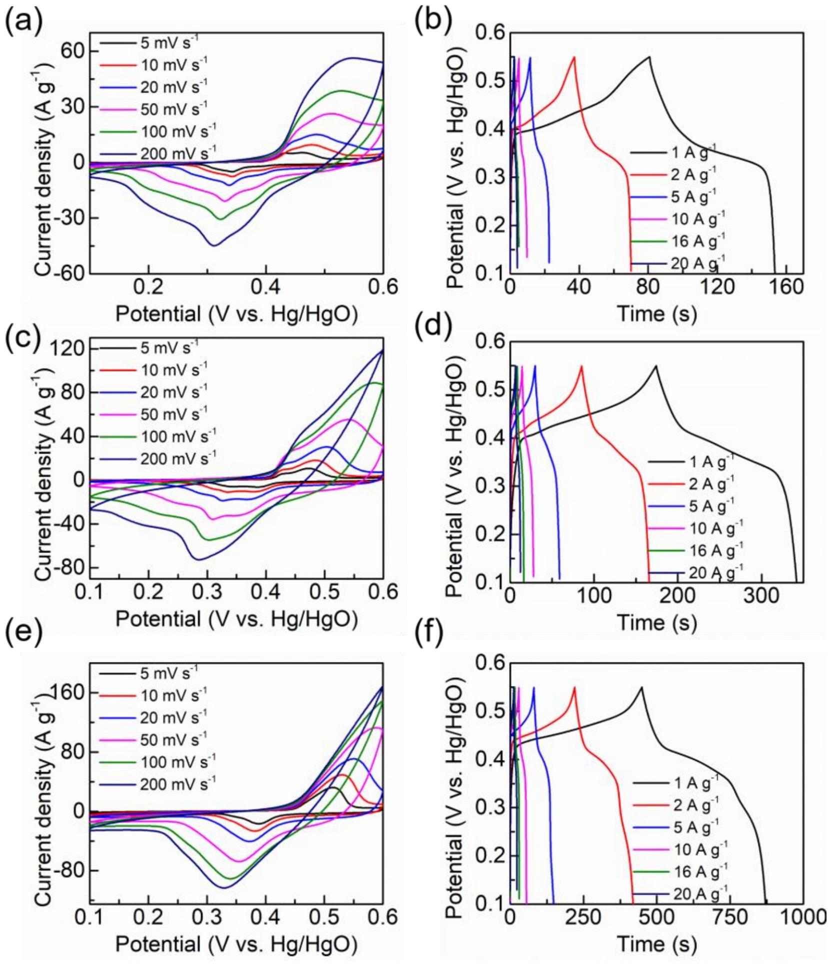

Fig. S15 CV curves **a** and GCD curves **b** of CCN-0-450; CV curves **c** and GCD curves **d** of CCN-0.2-400; CV curves **e** and GCD curves **f** of CCN-0.2-450

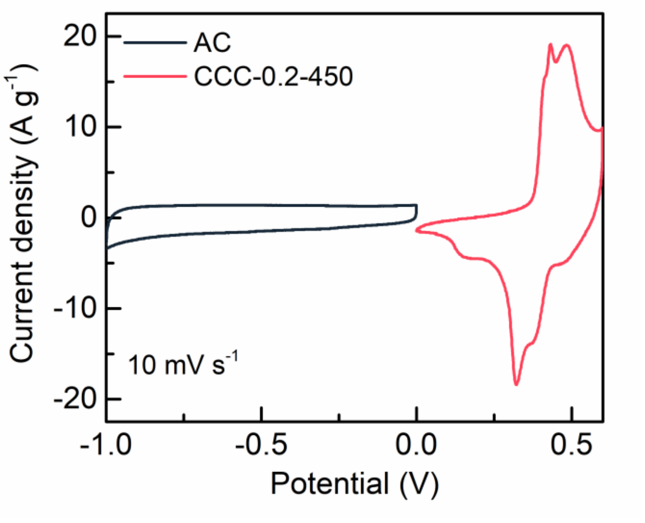

Fig. S16 Comparative CV curves of the positive and negative electrode

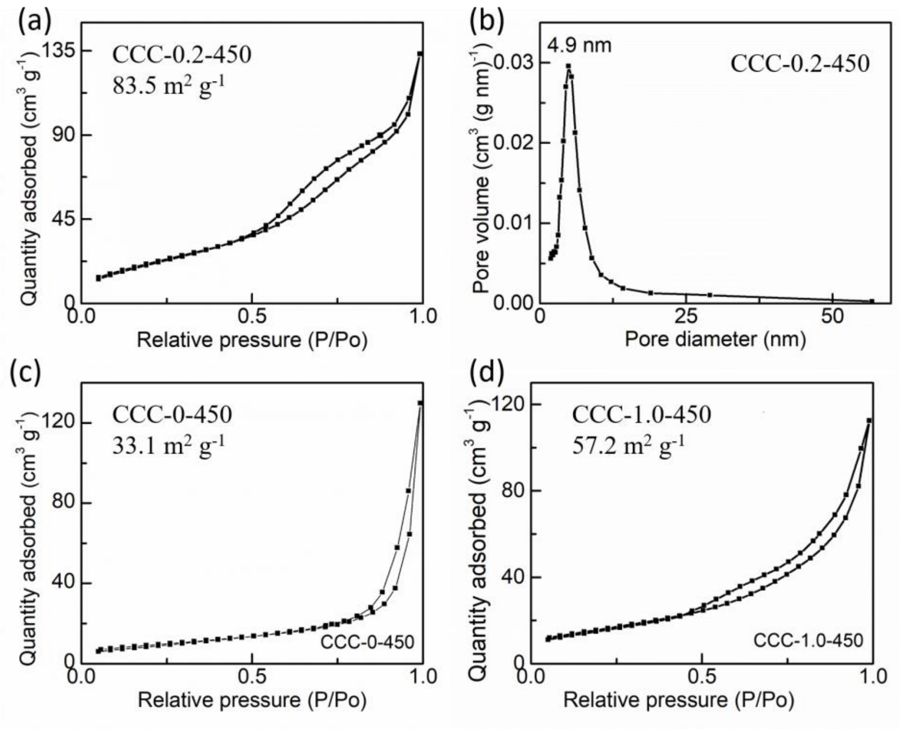

Fig. S17 Nitrogen adsorption-desorption isotherms: **a** CCC-0.2-450, **c** CCC-0-450 and **d** CCC-1.0-450; **b** pore-size distributions of CCC-0.2-450

References

[S1] N. Tang, W. Wang, H. You, Z. Zhai, J. Hilario et al., Morphology tuning of porous CoO nanowall towards enhanced electrochemical performance as supercapacitors electrodes. Catal. Today **330**, 240-245 (2019). https://doi.org/10.1016/j.cattod.2018.03.024

[S2] G. Li, M. Chen, Y. Ouyang, D. Yao, L. Lu et al., Manganese doped Co_3O_4 mesoporous nanoneedle array for long cycle-stable supercapacitors. Appl. Surf. Sci. **469**, 941-950 (2019). https://doi.org/10.1016/j.apsusc.2018.11.099

[S3] T. Chen, S. Li, J. Wen, P. Gui, Y. Guo et al., Rational construction of hollow core-branch $CoSe_2$ nanoarrays for high-performance asymmetric supercapacitor and efficient oxygen evolution. Small **14**(5), 1700979 (2018). https://doi.org/10.1002/smll.201700979

[S4] T. Liu, L. Zhang, W. You, J. Yu, Core-shell nitrogen-doped carbon hollow spheres/Co_3O_4 nanosheets as advanced electrode for high-performance supercapacitor. Small **14**(12), 1702407 (2018). https://doi.org/10.1002/smll.201702407

[S5] N. Zhang, X. Yan, J. Li, J. Ma, D. H. L. Ng, Biosorption-directed integration of hierarchical CoO/C composite with nickel foam for high-performance supercapacitor. Electrochim. Acta **226**, 132-139 (2017). https://doi.org/10.1016/j.electacta.2016.12.192

[S6] Y. Tan, Y. Liu, L. Kong, L. Kang, F. Ran, Supercapacitor electrode of nano-Co_3O_4 decorated with gold nanoparticles via in-situ reduction method. J. Power Sources **363**, 1-8 (2017). https://doi.org/10.1016/j.jpowsour.2017.07.054

[S7] Y. Zou, I. A. Kinloch, R. A. W. Dryfe, Mesoporous vertical Co_3O_4 nanosheet arrays on nitrogen-doped graphene foam with enhanced charge-storage performance. ACS Appl. Mater. Inter. **7**(41), 22831-22838 (2015). https://doi.org/10.1021/acsami.5b05095

[S8] C. Zheng, C. Cao, Z. Ali, J. Hou, Enhanced electrochemical performance of ball milled CoO for supercapacitor applications. J. Mater. Chem. A **2**(39), 16467-16473 (2014). https://doi.org/10.1039/C4TA02885F

[S9] Y. Jiang, C. He, S. Qiu, J. Zhang, X. Wang et al., Scalable mechanochemical coupling of homogeneous Co_3O_4 nanocrystals onto in-situ exfoliated graphene sheets for asymmetric supercapacitors. Chem. Eng. J. **397**, 125503 (2020). https://doi.org/10.1016/j.cej.2020.125503

[S10] Y. Ji, Y. Deng, F. Chen, Z. Wang, Y. Lin et al., Ultrathin Co_3O_4 nanosheets anchored on multi-heteroatom doped porous carbon derived from biowaste for high performance solid-state supercapacitors. Carbon **156**, 359-369 (2020). https://doi.org/10.1016/j.carbon.2019.09.064

[S11] Y. Tao, Y. Wu, H. Chen, W. Chen, J. Wang et al., Synthesis of amorphous hydroxyl-rich Co_3O_4 for flexible high-rate supercapacitor. Chem. Eng. J. **396**, 125364 (2020). https://doi.org/10.1016/j.cej.2020.125364

[S12] S. Yang, Y. Liu, Y. Hao, X. Yang, W. A. Goddard Iii et al., Oxygen-vacancy abundant ultrafine Co_3O_4/graphene composites for high-rate supercapacitor electrodes. Adv. Sci. **5**(4), 1700659 (2018). https://doi.org/10.1002/advs.201700659

[S13] Z. Shi, L. Xing, Y. Liu, Y. Gao, J. Liu, A porous biomass-based sandwich-structured Co_3O_4@carbon fiber@Co_3O_4 composite for high-performance supercapacitors. Carbon **129**, 819-825 (2018). https://doi.org/10.1016/j.carbon.2017.12.105
